# Implicit Attitudes Toward Psychotherapy and Explicit Barriers to Accessing Psychotherapy in Youths and Parent–Youth Dyads

**DOI:** 10.32872/cpe.7375

**Published:** 2022-09-30

**Authors:** Simone Pfeiffer, Ashley Huffer, Anna Feil, Tina In-Albon

**Affiliations:** 1 Department of Clinical Child and Adolescent Psychology and Psychotherapy, University of Koblenz-Landau, Landau, Germany; Philipps-University of Marburg, Marburg, Germany

**Keywords:** Implicit Association Test, psychotherapy, barriers, mental disorders, stigma, youths

## Abstract

**Background:**

Few studies have investigated implicit and explicit attitudes toward psychotherapy in youths (Study 1), although information about attitudes would improve interventions that aim to decrease barriers to accessing psychotherapy including parents (Study 2), who facilitate the help-seeking process of youths.

**Method:**

The Study 1 sample comprised 96 youths (14–21 years) and the Study 2 sample 38 parent–youth dyads. Differences in implicit attitudes regarding psychotherapy and a medical treatment were measured with the Implicit Association Test, and psychotherapy knowledge and self-reported barriers to psychotherapy were assessed with questionnaires. The actor-partner interdependence model was used to test the dyadic effects of implicit attitudes on explicit attitudes in parents and youths.

**Results:**

We did not find evidence for an implicit bias toward psychotherapy compared to a medical treatment, neither in youths, nor in parents. Self-reported barriers were a predictor for lower help-seeking intentions. Deficits in psychotherapy knowledge were more relevant in younger participants. Having a prior or current experience with psychotherapy and having a friend or family member with a prior or current experience with psychotherapy were predictors for better psychotherapy knowledge, but was not for lower barriers to accessing psychotherapy. Partner effects (degree to which the individual’s implicit attitudes are associated with explicit attitudes of the other dyad’s member) were not found.

**Conclusion:**

Specific deficits in psychotherapy knowledge should be addressed in interventions to lower barriers accessing psychotherapy. Parents should be included in interventions as a valuable resource to support youths in seeking psychotherapy for mental disorders.

## Highlights

- Implicit attitudes toward psychotherapy were comparable with attitudes toward a medical treatment.
- Youths reported explicit barriers to accessing psychotherapy and exhibited deficits in psychotherapy knowledge.
- Interventions aiming to reduce barriers to accessing psychotherapy should address specific knowledge deficits in youths.
- Parents should be included in interventions as a valuable resource to support youths in seeking psychotherapy for mental health problems.

Stigmatizing attitudes toward people with mental disorders as a barrier of help-seeking have been widely studied (Aguirre Velasco et al., 2020; Gulliver et al., 2010; Radez et al., 2021), however, there is a lack of studies investigating attitudes toward mental health care, especially psychotherapy, in youths. In their Mental Illness Stigma Framework, Fox and colleagues (2018) distinguish between experienced stigma and internalized stigma as a consequence of self-disclosure and anticipated stigma toward psychotherapy (the extent to which a person with a mental disorder expects to be the target of stereotypes, prejudice, or discrimination in the future), which is the focus of our study. In adult samples, negative attitudes toward mental health care use, especially the presence of stigma, low perceived efficacy of treatments, or the desire to handle the problem on their own, are the most common barriers to seek treatment for mental disorders (Andrade et al., 2014; Mojtabai et al., 2011; Van Voorhees et al., 2006). When asked specifically about psychotherapy, adults have reported mainly positive attitudes (Petrowski et al., 2014) yet also that they would be ashamed if neighbors and friends knew about the use of psychotherapy (Albani et al., 2013).

Most of those studies use explicit measures to assess attitudes toward mental health care, implicit measures however can elicit more spontaneous responses than explicit measures, whereas explicit measures are related to deliberative decisions about the conformation or rejection of attitudes. There is evidence that the assessment of implicit and explicit attitudes are distinct measures with a rather weak relationship and the need to consider (negative) attitudes as a multifaceted construct (Brauer et al., 2000). The combination of implicit and explicit measures to assess barriers toward psychotherapy might be promising in capturing the complexity of attitudes toward psychotherapy.

Studies investigating implicit attitudes toward mental disorders using the Implicit Association Test (IAT) found that adults reported a more negative implicit attitude toward people with mental disorders than toward people with a physical illness (González-Sanguino et al., 2020; Teachman et al., 2006). O’Driscoll et al. (2012) found higher stigmatization for a vignette describing an individual with depression compared to an individual with attention-deficit/hyperactivity disorder in boys, but not in girls. In a sample of young adults, depression was associated with more implicit, but not explicit, negative attitudes compared to a physical illness (Monteith & Pettit, 2011). Little is known about the effects of implicit attitudes toward psychotherapy with regard to low treatment rates for mental disorders in youths and negative attitudes toward people with mental disorders.

Negative attitudes are influenced by gender, age, and personal experience with mental disorders and help seeking. In men compared to women, there is evidence of lower help-seeking intentions for mental health problems (Addis & Mahalik, 2003; Oliver et al., 2005; Petrowski et al., 2014) and less mental health knowledge (Farrer et al., 2008). Boys compared to girls have reported higher mental health stigma and less willingness to use mental health services (Calear et al., 2011; Chandra & Minkovitz, 2006; Gonzalez et al., 2005). Further, there is evidence that higher age is associated with higher mental health knowledge and a less stigmatizing attitude (Farrer et al., 2008; Swords et al., 2011) and more acceptance of peers with mental disorders (Swords et al., 2011).

A prior experience with a mental disorder or psychotherapy, or familiarity with someone who has a mental disorder, which is associated with less stigmatizing attitudes in children, youths, and adults (Bellanca & Pote, 2013; Griffiths et al., 2008; Sandhu et al., 2019) may be seen as protective factors for stigmatizing attitudes. The identification of possible risk factors (e.g., gender, specific age group) might enable to develop age- or gender- tailored interventions to reduce barriers toward psychotherapy or interventions, which include contact to a person with a prior experience of psychotherapy.

## Parental Role in Attitudes Toward Psychotherapy

Children and youths often prefer informal sources of help for mental disorders, such as parents (Rickwood et al., 2005). When assessing attitudes toward psychotherapy in youths, it is important to include parental attitudes, as they are important key gatekeepers to mental health care access and as they also report negative attitudes toward mental health care (Reardon et al., 2017). Despite youths’ growing autonomy, their decision to seek professional help for mental health problems is highly influenced by their parents (Gulliver et al., 2012; Rickwood et al., 2005; Ryan et al., 2015), who are often the first to recognize mental health problems in their children and the need for help. In their model for a parent-mediated pathway to mental health services for adolescents, Logan and King (2001) emphasized the important role of parent’s attitudes toward mental health care in the help-seeking process of their child.

Youths’ willingness to seek help is higher when they think their parents support the use of mental health services (Chandra & Minkovitz, 2006; Wahlin & Deane, 2012) and lower when they think their parents would be ashamed of them because of their mental health problems (Moses, 2009). Little is known about whether youths report similar attitudes toward mental health to those of their parents. There is evidence that youths seem to agree with their parents’ evaluation of the helpfulness of mental health services (Jorm & Wright, 2007) but that parents and youth differ in their knowledge and explicit attitudes toward mental disorders, with youths showing higher stigma scores and less mental health knowledge compared to their parents (Lorona & Miller-Perrin, 2016). There is, however, a lack of research evaluating implicit and explicit attitudes toward psychotherapy and their relationship which each other in parent–youth dyads.

## Aims of the Study

In Study 1, the first aim was to compare implicit attitudes toward psychotherapy with attitudes toward a medical treatment using the IAT (Greenwald et al., 1998). We decided to contrast psychotherapy with a medical treatment to control for attitudes that are generally associated with help-seeking behavior for (mental) health problems (e.g., being confronted with symptoms of [psycho]pathology). In this study, we were particularly interested if attitudes differ about consulting a pediatrician or general practitioner for health-related symptoms versus a psychotherapist for mental health problems. The second aim was to assess explicit barriers to accessing psychotherapy as well as psychotherapy knowledge in youths and their influence on help-seeking intentions, for which we used a self-report questionnaire. We hypothesized that higher positive implicit attitudes toward psychotherapy compared to a medical treatment, higher psychotherapy knowledge, and lower explicit barriers to accessing psychotherapy would be associated with higher help-seeking intentions. As there are gender and age differences in attitudes toward mental health care, we evaluated if male participants reported more negative attitudes toward psychotherapy than female participants and if negative attitudes toward psychotherapy decrease with age. The analysis of differences between different education levels were analyzed exploratively. We further expected fewer barriers to accessing psychotherapy and better psychotherapy knowledge in participants with a prior or current experience with psychotherapy and in those who had a friend or family member involved in psychotherapy. In Study 2, we investigated parents and youths’ implicit and explicit attitudes (barriers) toward psychotherapy and their relationship considering the dyadic structure of the data.

## Study 1

### Method

#### Participants

A total of 96 youths between the age of 14 and 21 years participated in this study (*M* = 18.4 years, *SD* = 2.1). In this sample, 68% self-identified as female and 32% as male. In terms of education, 69% attended a secondary school, 21% a university, and 10% a vocational school. Nineteen percent had a prior or current experience with psychotherapy and 72% rated the experience as positive. In all, 72% were familiar with someone having sought or seeking psychotherapy and 78% rated that person’s experience as positive. Participants were recruited online via social media and in local secondary schools. The inclusion criterion to participate in the study was being between 14 and 21 years of age. We chose the age of 14 years because youth can participate in studies without a written parental consent and 21 years as this is the age limit for child and adolescent psychotherapy in Germany.

#### Measures

##### Implicit Association Test (IAT)

The IAT (Greenwald et al., 1998) is a computerized dichotomous categorization task measuring association strengths between concepts and attributes. The outcome measure is response time (milliseconds), with shorter latencies indicating stronger automatic associations of concepts with the stimulus group. The key IAT assumption is that participants show faster reaction times when stimuli are paired in ways that are consistent versus inconsistent with well-learned automatic associations, that is, implicit biases. The IAT is a relative assessment; that is, evaluations of one group are compared with evaluations of a second group (Greenwald et al., 1998).

Regarding the concepts, psychotherapy and a medical treatment were compared using words as stimuli. Psychotherapy was primed with words *psychotherapist, psychotherapist’s practice, psychological conversation, psychology,* and *children’s and adolescents’ psychotherapist*; medical treatment was primed with *general practitioner, general practitioner’s practice, medical exam, medicine,* and *pediatrician*. We chose positive and negative attributes associated with psychotherapy (Maier et al., 2014). Positive attributes were *professional, effective, trustworthy, competent,* and *meaningful* and negative attributes were *unprofessional, ineffective, untrustworthy, incompetent,* and *meaningless.* The categorization of concepts and attributes was checked in advance with four youths that correctly assigned the priming words to the concepts and the attributes.

In our pilot study, we evaluated time-differences for concepts and attributes and did not find differences between the concept “medical treatment” and “psychotherapy”, *t*(8)= 0.87, *p* = .38 or between the attributes “positive” and “negative”, *t*(8)= 0.29, *p* = .77.

The IAT was constructed with online-survey software, a valid and reliable approach (Carpenter et al., 2019) using Sosci-Survey (Leiner, 2019). The IAT consists of seven “blocks” (sets of trials) and in each block, participants see a stimulus (word) on the screen. Stimuli represent concepts (medical treatment or psychotherapy) or attributes (positive - negative). When stimuli appear, the participant “sorts” the stimulus as rapidly as possible by pressing with either their left or right hands on the keyboard (the “E” an “I” keys). The sides with which one should press are indicated in the upper left and right corners of the screen. If the target word was a member of the category listed on the left side of the screen, the participants were to respond with the *E* key. If the target word was a member of the category listed on the right side of the screen, the participants were to respond with the I key. A correct response was required before continuing to the next slide and response latencies were recorded from the presentation of the stimulus to the correct response. The initial pairing of concepts and attributes was counterbalanced across participants. The interstimulus interval was 300 ms. Block 1 is used to practice the two categories; participants distinguished between the target categories of medical treatment and psychotherapy. The priming words were presented in a random order and were distinguished by designated keys on the left or right side of the keyboard (e.g., left for medical treatment, right for psychotherapy). Block 2 is used to practice the attributes (positive vs. negative); participants distinguished positive attributes from negative attributes presented on the screen. Block 3 is the first pairing of categories and attributes; participants distinguished between medical treatment and positive attributes versus psychotherapy and negative attributes by pressing the designated keys. Block 4 repeats the Block 3 pairings. In Block 5, responses to the positive attributes and negative attributes are reversed. Both Blocks 6 and 7 are test blocks that consist of the second category and attribute pairing; participants distinguished between medical treatment and negative attributes versus psychotherapy and positive attributes. The order in which each pairing was presented and associated with the key on the right or left side of the keyboard (Blocks 3 and 4 vs. Blocks 6 and 7) was randomized.

##### Barriers to Accessing Psychotherapy

To assess explicit barriers to accessing psychotherapy, we developed a self-report questionnaire. First, we conducted a literature review on attitudes toward psychotherapy, from which we drew 13 statements (Table 1). In a pilot study, youths (*n* = 9) rated the comprehensibility of the statements on a 6-point Likert scale (1 = *totally disagree*, 6 = *totally agree*). To explore the factor structure, the 13 items were subjected to an exploratory analysis with oblique rotation. The Kaiser–Meyer–Olkin (KMO) measure verified the sampling adequacy for the analysis (KMO = 0.85). Bartlett’s test of sphericity, χ^2^(78) = 406.72, *p* < .001, indicated that the correlation structure was adequate for factor analysis. A maximum likelihood factor analysis with a cutoff point of .40 and Kaiser’s criterion of eigenvalues greater than 1 yielded a one-factor solution as the best fit for the data, with the root mean square of residuals = 0.06, the root mean square error of approximation = 0.08, and the Tucker–Lewis Index = 0.96, an acceptable value considering it is over 0.9. One item (“I would prefer other treatment options than psychotherapy”) did not load on the factor and was excluded from further analyses. Internal consistency was good with McDonald’s omega = 0.88.

##### Help-Seeking Intention and Familiarity With Psychotherapy

We asked participants to rate their anticipated probability of initiating psychotherapy in the event of serious mental health problems (0–100%) and to indicate if they had current or past experience with psychotherapy themselves, and if they had a friend or family member who had current or past experience with psychotherapy. They also rated whether the experience (or reported experience) was positive (1) or negative (0) using a dichotomous item. The items were taken from a previous study (Pfeiffer & In-Albon, 2022).

##### Psychotherapy Knowledge

We assessed psychotherapy knowledge with a self-developed questionnaire with 11 statements based on a literature search (e.g., knowledge about the professional confidentiality, the nonpsychoanalytical setting, multifactorial causes of mental disorders), which are listed in Table 3. Six licensed psychotherapists rated the statements for correctness and we made adjustments in two steps. First, we used Fleiss’s kappa to measure interrater reliability. We found κ = 1 (perfect agreement) for nine of the items and lower kappas for Item 1 (κ = .5) and Item 5 (κ = .33). These two items were then revised and rated again, resulting in perfect interrater agreement of κ = 1 for all items. Participants were asked to indicate if the statements were true or false or to indicate that they did not know the answer (“I don’t know”). Before conducting the pilot-study, we conducted a pretest with four youths who rated the statements for sufficient feasibility, which lead to the revision of one item because of the use of professional jargon.

#### Procedure

The local ethics committee approved the study (reference number: LEK_262). Parents and youths were informed about the content and aims of the study. Written consent in accordance with the Declaration of Helsinki from parents and youths was mandatory for participants. We conducted a pilot study in advance with *n* = 9 youths to test the feasibility of the study design.

Parents and youths received a link and a QR code to participate in the online study. Study duration was 20-25 minutes. Researcher were available to answer questions during the study. Participants did not receive compensation.

#### Data Processing and Statistical Analyses

Statistical analyses were conducted with R (version 4.03). For the evaluation of implicit attitudes, we used the improved D score (Greenwald et al., 2003), which measures the strength and direction of the implicit association. We included all participants who completed the study. Reaction times faster than 300ms and slower than 10 seconds were excluded from further evaluation (*n* = 1).

Positive improved D scores suggest a stronger association between medical treatment and positive attributes than psychotherapy and negative attributes. Negative D scores suggest that the association between psychotherapy and positive attributes is higher compared to medical treatment and negative attributes.

For psychotherapy knowledge, we calculated the total score using the number of correct answers (correct answer = 1; wrong answer or “I don’t know” = 0). Exploratively, we examined if implicit attitudes toward psychotherapy and barriers to accessing psychotherapy as well as psychotherapy knowledge varied with gender, age, or education using a multivariate analysis of variance and multiple regression analysis. An a- priori power analysis was conducted with g*Power (Faul et al., 2007). For the MANOVA a sample size of *n* = 84 is necessary to detect a small effect, *f^2^* = 0.10, 1-ß = 0.95, α = 0.05. For the multiple regression analysis, a sample size of *n* = 70 is necessary to detect a small effect, *f*^2^ = 0.10, 1-ß = 0.95, α = 0.05. Multiple regressions were calculated to determine if implicit attitudes toward psychotherapy and barriers to accessing psychotherapy as well as psychotherapy knowledge predict higher help-seeking intentions. Multiple regressions were also calculated to determine if a prior or current experience with psychotherapy or familiarity with someone seeking psychotherapy predicts fewer negative implicit attitudes toward psychotherapy, fewer barriers to accessing psychotherapy, and better psychotherapy knowledge. We used dummy variables with 1= prior or current experience and 0= the absence of a prior or current experience.

### Results

#### IAT

We did not find evidence for a stronger association neither for positive nor for negative attributes with psychotherapy compared to a medical treatment with an improved D score of *M* = 0.09 (*SD* = 0.41).

#### Barriers to Accessing Psychotherapy

The descriptive statistics regarding explicit barriers to accessing psychotherapy indicate an overall moderate agreement with barriers (Table 1).

**Table 1:** Barriers to Accessing Psychotherapy in Study 1 (Youths) and Study 2 (Youth–Parent Dyads)

Item	Study 1 Youths	Study 2 Youths	Study 2 Parents
	*M* (*SD*)	*M* (*SD*)	*M* (*SD*)
1. I would be afraid that psychotherapy would make my problems worse.	2.45 (1.18)	2.79 (1.42)	2.34 (1.28)
2. I would be concerned that my problems would not be treated confidentially.	3.03 (1.57)	2.29 (1.35)	2.26 (1.37)
3. I would think that starting psychotherapy costs money and is too expensive.	2.57 (1.50)	2.71 (1.56)	2.21 (1.18)
4. I would be afraid that the psychotherapist would judge me or think something bad about me.	2.50 (1.47)	2.05 (1.14)	1.63 (0.91)
5. I would be afraid that the psychotherapist would admit me to a psychiatric facility against my will.	3.23 (1.48)	2.84 (1.41)	2.26 (1.18)
6. I would think a psychotherapist doesn’t understand my problems.	2.95 (1.37)	3.45 (1.78)	3.63 (1.75)
7. I had negative previous experiences with psychologists/psychotherapists.	2.94 (1.51)	2.26 (1.41)	1.74 (0.95)
8. My parents/my environment would not support me in starting psychotherapy.	2.90 (1.41)	2.16 (1.20)	4.55 (1.35)
9. I would be concerned that starting psychotherapy would say something bad about my family.	3.28 (1.55)	2.58 (1.18)	2.34 (1.02)
10. I would be afraid of not knowing what happens during psychotherapy.	3.39 (1.52)	2.45 (1.25)	2.39 (1.20)
11. I would be afraid to talk about my problems with a psychotherapist.	3.06 (1.41)	3.05 (1.45)	3.05 (1.63)
12. I wouldn’t think psychotherapy would help.	2.58 (1.47)	3.47 (1.61)	4.34 (1.65)
Total score	2.60 (0.88)	2.67 (0.84)	2.94 (0.49)

*Note.* Items were rated on a 6-point Likert scale (1 = *totally disagree*, 6 = *totally agree*).

#### Help-Seeking Intention and Familiarity With Psychotherapy

The intention to seek psychotherapy in the event of mental health problems had a median of 60% (range 0–100%). Multiple linear regressions indicated an overall effect for implicit attitudes, explicit barriers and psychotherapy-knowledge as predictors for help-seeking intentions, *R*^2^ = .23, *F*(3, 91) = 8.91, *p* < .001, with a significant effect in explicit barriers to accessing psychotherapy as predictor for lower help-seeking intention, *b* = -1.20, ß = -0.45, CI 95% [-1.71, -0.69], *SE*_*b* = 0.25, *t*(91) = -4.72, *p* < .001, whereas implicit attitudes, *b* = -0.43, CI 95% [-0.63, 1.49], ß = 0.08, *SE*_*b* = 0.53, *t*(91) = 0.81, *p* =.42 and psychotherapy knowledge, *b* = 0.07, CI 95% [-0.11, 0.26], ß = 0.08, *SE*_ *b* = 0.09, *t*(91) = 0.82, *p* = .41 were not associated with higher or lower help-seeking intentions.

Multiple regression analysis were conducted to investigate if a prior or current experience of psychotherapy or familiarity with a person seeking psychotherapy are predictors of levels in implicit attitudes (Model 1), *R*^2^ = .00, *F*(2, 92) = 1.08, *p* = 0.34, barriers toward psychotherapy (Model 2), *R*^2^ = .00, *F*(2, 93) = 0.48, *p* = 0.61, and psychotherapy knowledge (Model 3), *R*^2^ = .22, *F*(2, 93) = 14.21, *p* < .001, and are reported in Table 2.

**Table 2:** Results From Multiple Regression Analysis for Prior or Current Experience of Psychotherapy and Familiarity With People Seeking Psychotherapy as Predictors for Implicit Attitudes (Model 1), Explicit Barriers (Model 2), and Psychotherapy Knowledge (Model 3)

Estimates	*B*	*SE*	*Beta* (β)	*t*	*p*
Model 1 (implicit attitudes)					
intercept	0.14	0.08	0.00	1.75	.08
experience_pt	0.15	0.71	-0.14	-1.35	.18
familiarity	-0.04	0.09	-0.04	-0.42	.67
Model 2 (explicit attitudes]					
intercept	2.70	0.17	0.00	15.65	< .001
experience_pt	-0.20	0.23	-0.09	-0.85	0.40
familiarity	-0.08	0.20	-0.04	-0.38	0.70
Model 3 (psychotherapy knowledge)					
intercept	4.33	0.43	0.00	9.98	< .001
experience_pt	2.00	0.59	0.31	3.40	< .001
familiarity	1.87	0.51	0.33	3.66	< .001

*Note.* experience_pt = prior or current experience with psychotherapy.

#### Psychotherapy Knowledge

Participants’ psychotherapy knowledge is reported in Table 3.

**Table 3:** Percentages of Correct, Incorrect, and “I Don’t Know” Answers for Psychotherapy Knowledge Items

Item	Correct answer (%)	Incorrect answer (%)	I don’t know (%)
1. The costs of psychotherapy are usually covered by health insurance.	47	13	41
2. During psychotherapy, the patient is usually lying on a couch.	77	5	18
3. In a psychotherapy patients take an active part in the decision making concerning the psychotherapy process.	58	7	34
4. Mental illnesses often manifest as physical symptoms, e.g., abdominal pain and headaches.	67	14	20
5. Over 40% of all people meet the criteria of a mental disorder during their lifetime.	49	7	44
6. The origin of mental disorders is exclusively genetic.	81	4	15
7. The effectiveness of psychotherapy is proven by scientific studies.	53	6	41
8. From the age of 15, I am allowed to start psychotherapy without the consent of my parents.	17	6	77
9. A psychotherapist is allowed to speak with my parents about the content of my psychotherapy without my consent.	72	7	21
10. Health insurance pays for trial sessions to find out if I want to work with the therapist.	34	5	60
11. A therapist helps me become an expert on my own problems.	50	11	39

*Note.* Percentages do not sum up to 100% due to rounding.

#### Age, Gender, and Education Differences in Implicit Attitudes, Explicit Barriers, and Psychotherapy Knowledge

Contrary to our expectations, we did not find gender differences, *F*(3, 93) = 2.09, *p* = .13, or differences between education levels, *F*(3, 93) = 0.15 *p* = .87, in implicit attitudes toward psychotherapy, explicit barriers to accessing psychotherapy, or psychotherapy knowledge as a result of a MANOVA. We conducted a single predictor regression analysis to examine if age is associated with implicit attitudes, explicit barriers, and psychotherapy knowledge and found a significant overall effect, *R*^2^ = .12, *F*(3, 91) = 3.99, *p* < .001.

Higher age was associated with higher psychotherapy knowledge, *b* = 0.29, CI 95% [0.12, 0.47], ß = 0.35, *SE*_*b* = 0.09, *t*(91) = 3.42, *p* < .001, however age was not a predictor for implicit attitudes, *b* = 0.32, CI 95% [-0.68, 1.32], ß = 0.06, *SE*_*b* = 0.50, *t*(91) = 0.63, *p* = .53, or explicit barriers seeking psychotherapy, *b* = 0.31, CI 95% [-0.17, 0.79], ß = 0.13, *SE_b* = 0.24, *t*(91) = 1.30, *p* = .20.

## Study 2

### Method

#### Participants

In Study 2, 38 parent–youth dyads participated. The youths (*M*_age_ = 18.5 years, *SD* = 2.0, range: 14–21) had not participated in Study 1. Here, 68% identified themselves as female. In terms of education, 37% attended a secondary school, 57% a university, 6% a vocational school.

The parent sample had an age range of 38–62 years (*M* = 49.6 b, *SD* = 5.7) and 76% identified themselves as female. Twenty-nine percent had a prior or current experience with psychotherapy and 64% of them rated the experience as positive. Seventy-nine percent were familiar with people having sought or seeking psychotherapy, with 77% rating the reported experience as positive.

#### Measures

The IAT and explicit barriers measure were identical to those in Study 1. Regarding help seeking, youths were asked if they thought they would receive support from their parents, and parents were asked if they would seek support from their close network.

#### Procedure

The procedure was identical to that in Study 1. Parents and youths were asked to create the same code to assign the parent-youth dyad.

#### Data Processing and Statistical Analysis

Statistical analyses were conducted with R (version 4.03). The data treatment was identical to Study 1. Three participants were excluded from further analysis because their codes did not match with a corresponded code. Descriptive statistics and Welch sample *t*-tests for implicit attitudes and barriers toward psychotherapy between youths and parents were calculated.

Considering the dyadic structure of the data we conducted an actor-partner-interdependence model (APIM) using the lavaan package for structural equation modelling (SEM). APIMs are useful for exploring the dynamic interplay between relational partners, in our case parents and youths (Kenny, Kashy, & Cook, 2006). This model is based on the fact that the scores within the same dyad are not independent but instead are more similar than the scores of two individuals, who are not in the same dyad. The APIM is useful to determine how parameters (explicit and implicit attitudes) among youth and parent are influenced by not only internal factors but also factors related to the other member of the dyad. Structural equation modeling simultaneously examines both paths in the APIM: two actor effects (i.e., each person’s implicit attitudes regressed on his or her own explicit attitudes) and two partner effects (i.e., each person’s implicit attitudes regressed on the other person’s explicit attitudes).

### Results

Consistent with the results of Study 1, we did not find evidence for a stronger association neither for positive nor for negative attributes with psychotherapy compared to a medical treatment with an improved D score of *M* = 0.04 (*SD* = 0.47) for youths and *M* = 0.12 (*SD* = 0.51). Means and standard deviations for barriers toward psychotherapy are reported in Table 2. Analyzing mean scores, parents and youths did differ in explicit attitudes, *t*(44) = 2.88, *p* = .01, but not in their implicit attitudes, *t*(73) = 0.70, *p* = .46.

**Figure 1:**
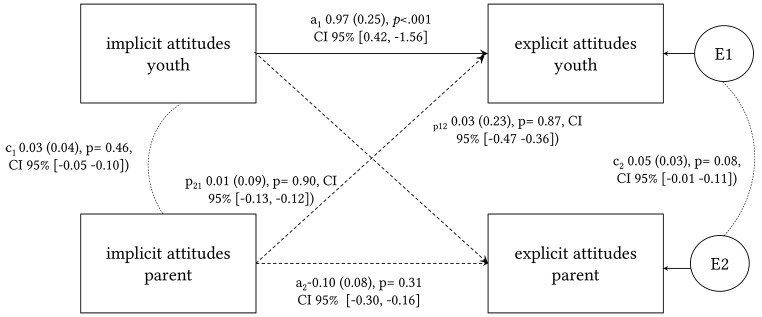
Path Diagram of the Actor-Partner-Interdependence Model (APIM) With Implicit Attitudes Being a Predictor for Explicit Attitudes *Note.* a1, a2 = actor effect; p12, p21 = partner effect; c1 = covariance of implicit attitudes between parent and youth; c2 = residual non-independence of explicit attitudes. ***p < .001.

The results of the APIM analysis for explicit attitudes and implicit attitudes are set out in Figure 1. The goodness of fit measures were good with χ^2^(*N* = 38, 6) = 18.68, *p* = .01, CFI = 1.00, TLI = 1.00, RMSEA = 0.00, SRMR = 0.00 with except for the Chi-square test, which is however sensitive to sample size. The actor effect for youths was significant with implicit attitudes being a predictor for explicit attitudes in youths, which has not been the case for the parent sample. There was no evidence for a partner effect.

## General Discussion

In contrast to the higher stigmatization of mental disorders when compared to physical illnesses (González-Sanguino et al., 2020; Teachman et al., 2006), psychotherapy was not more stigmatized when evaluating implicit attitudes in comparison to a medical treatment. This result is in line with findings of mainly positive explicit attitudes toward psychotherapy in a general nonclinical adult sample (Petrowski et al., 2014). The youth sample in the present study did, however, agree with explicit specific barriers to accessing psychotherapy, probably reflecting more negative attitudes when confronted with the idea of actual help seeking instead of psychotherapy in general, which is consistent with findings in adults (Albani et al., 2013). Higher barriers to accessing psychotherapy were also, as expected, associated with lower help-seeking intentions, which is consistent with findings in adult samples regarding attitudes toward mental health care (Andrade et al., 2014; Mojtabai et al., 2002; Van Voorhees et al., 2006). Regarding psychotherapy knowledge, the results were mixed (see Table 2) revealing deficits in psychotherapy-knowledge. Interventions aiming to increase mental health knowledge should include information about the setting and general framework of psychotherapy to facilitate the decision to access it. In our sample, higher psychotherapy knowledge was not associated with higher help-seeking intentions, but the interpretation of the results is limited by the high number of youths indicating knowledge deficits.

We did not find gender differences for implicit attitudes toward psychotherapy, explicit barriers to accessing psychotherapy, or psychotherapy knowledge. In contrast to other studies that found gender differences for mental health knowledge and attitudes toward mental health care use (Chandra & Minkovitz, 2006; Gonzalez et al., 2005), we focused specifically on psychotherapy, which might represent a different construct from mental health care in general that includes treatment in inpatient settings and psychopharmacotherapy. Overall, there are few studies evaluating gender differences in this field of research in youths.

The results indicate less psychotherapy knowledge in younger youths compared to participants with older youths, which is consistent with other findings (Farrer et al., 2008; Swords et al., 2011). Barriers to accessing psychotherapy seemed to increase with age and were associated with lower help-seeking intentions. More research is necessary to determine age-related factors to improve interventions aiming to lower barriers to accessing psychotherapy in specific age groups.

Having a prior or current experience with psychotherapy and being familiar with someone with a prior or current experience with psychotherapy were predictors for higher psychotherapy-knowledge, but surprisingly not with fewer implicit and explicit attitudes toward psychotherapy. However, the interpretation of this result is limited, as we had only a small sample of those seeking psychotherapy and a lack of further information (e.g., number of sessions).

When we analyzed the data from parent–youth dyads, we found evidence of similar implicit attitudes toward psychotherapy. Comparable with Study 1, psychotherapy was not more highly stigmatized than medical treatment in youths as well as in parents. The dyadic analyses for implicit and explicit attitudes based on the APIM revealed an actor effect for youths with implicit attitudes being a predictor for explicit attitudes, meaning that higher improved-d scores (a stronger association between medical treatment and positive attributes than psychotherapy and negative attributes) were predictors for more negative explicit attitudes. This might be evidence for a higher congruency in youths implicit and explicit attitudes, whereas parents’ explicit attitudes were not predicted by their implicit attitudes. We did not find partner effects for parents’ implicit attitudes being a predictor for youths’ explicit attitudes and vice versa. The covariance between youths and parent implicit and explicit attitudes were also non-significant. To sum up, parental explicit and implicit attitudes toward psychotherapy seem to be independent from youths` explicit and implicit attitudes with youths reporting less explicit barriers than parents. This might be due to a higher awareness of mental disorders and their treatment by exposure to interventions (e.g., in schools) aiming to increase mental health knowledge and decrease stigmatizing attitudes toward people with mental disorders (Reavley & Jorm, 2012). These results also indicate that interventions aiming to decrease barriers of help-seeking for mental disorders are well invested in youths who build their attitudes more and more independently of their parent’s attitudes when transitioning into adulthood. However, these results also emphasize the need to include parents in interventions to lower barriers to seeking psychotherapy, as they play an important role in supporting their children during the professional help-seeking process (Logan & King, 2001). Lowering barriers to accessing psychotherapy in parents might increase recognition of their child’s need for help and encourage them to search for professional help in the event of mental health problems. In conclusion, the results suggest that interventions or campaigns promoting a positive image of psychotherapy might be less relevant than intervention focusing on the reduction of specific barriers toward psychotherapy and deficits in psychotherapy-knowledge. There is evidence that parents should be included in interventions as a valuable resource to support youths in the help-seeking process for a mental disorder.

### Limitations

There are some limitations with regard to the use of the IAT to assess implicit attitudes toward psychotherapy (see Meissner et al., 2019). We did not assess whether a negative evaluation of psychotherapy predicts actual help-seeking behavior, as we assessed only help-seeking intentions. We also chose to contrast psychotherapy with a medical treatment, assessing the relative strength of the associations with the attributes. For this reason, we do not know if psychotherapy is perceived as positive, negative or neutral, the only knowledge we have is that psychotherapy is not perceived more negatively compared to a medical treatment. Future studies might choose different implicit measures, for example, a single IAT (Teige-Mocigemba et al., 2008) to evaluate the association of psychotherapy with attributes independent of a reference to a medical treatment.

The age differences might also pose problems as older participants may have very different needs and knowledge compared to younger participants. The gatekeeper role accessing mental health treatment might vary with age and further analyses are necessary to determine to which extend parents are still important gatekeeper for youths in their transition to adulthood. Although youths in emerging adulthood get more and more autonomous, parents still play an important role in their life might be an important source to discuss sensitive topics (Jiang et al., 2017), for example mental health problems and treatment use. In the parent sample, we had higher participation of mothers (76%) compared to fathers. The sample size was low for dyadic data analysis with an insufficient power of 0.7 to detect an actor effect in youths and a power of 0.05 to detect a partner effect for parents, whereas the power was good with 0.8 to detect an actor effect in parents and 1.00 to detect a partner effect in youths. Therefore, analysis should be conducted with a larger sample size.
